# Biochemical Recurrence in High-Risk Localized Prostate Cancer: A Comparison of Laparoscopic Prostatectomy and External Radiotherapy

**DOI:** 10.3390/medicina61050928

**Published:** 2025-05-20

**Authors:** Lubos Rybar, Patrik Hesko, Michal Miko, Peter Bujdak, Stefan Harsanyi

**Affiliations:** 1 Urology Department, St. Cyril and Methodius Hospital, University Hospital Bratislava, Antolska 11, 851 07 Bratislava, Slovakia; lubosrybar@gmail.com (L.R.);; 2 Department of Urology, Faculty of Medicine, University Hospital Bratislava, Comenius University in Bratislava, 833 05 Bratislava, Slovakia; 3 Institute of Histology and Embryology, Faculty of Medicine, Comenius University in Bratislava, Sasinkova 4, 811 08 Bratislava, Slovakia; 4 Institute of Medical Biology, Genetics and Clinical Genetics, Faculty of Medicine, Comenius University in Bratislava, Sasinkova 4, 811 08 Bratislava, Slovakia

**Keywords:** prostate cancer, biochemical recurrence, BCR, LRP, EBRT, iPSA

## Abstract

*Background*: This retrospective study evaluated and compared oncological outcomes in patients with localized prostate cancer treated either by laparoscopic radical prostatectomy (LRP) or by external beam radiotherapy (EBRT) combined with androgen deprivation therapy (ADT). The primary aim was to identify predictors of biochemical recurrence (BCR) and to assess recurrence-free survival. *Subjects and methods*: A total of 107 patients diagnosed with localized prostate cancer and treated between 2016 and 2023 were included in the analysis. Of these, 61 patients underwent LRP, and 46 patients received EBRT+ADT. The median follow-up period was 60 months for the LRP group (IQR 24–72) and 66 months for the EBRT group (IQR 49.5–72). Biochemical recurrence (BCR) was defined as a PSA level > 0.2 ng/mL after LRP or an increase > 2 ng/mL above nadir following EBRT. Kaplan–Meier survival curves, log-rank tests, Pearson’s chi-square, and Cox regression models were used to evaluate outcomes and identify predictors of recurrence, with significance set at *p* < 0.05. *Results*: Biochemical recurrence occurred in 21 (34.4%) of LRP patients and 10 (21.7%) of EBRT patients. The five-year BCR-free survival was 40 (65.6%) patients in the LRP group and 33 (71.7%) for EBRT, with a trend toward improved outcomes in the EBRT group that approached statistical significance (log-rank *p* = 0.089). Median time to recurrence was 30 months for LRP (IQR 12.75–60) and 48 months for EBRT (IQR 30–60). Predictive analysis revealed that in the LRP group, higher ISUP grade at biopsy (*p* = 0.001), advanced pathological stage (*p* < 0.001), positive surgical margins (*p* < 0.001), and intermediate initial PSA levels (10–20 ng/mL; *p* = 0.080) were associated with increased risk of BCR. No independent predictors of recurrence were identified in the EBRT group. *Conclusions*: Both LRP and EBRT+ADT provide effective cancer control with similar five-year BCR-free survival. However, LRP was associated with a higher recurrence rate, particularly among patients with intermediate-risk features such as iPSA 10–20 ng/mL, high ISUP grade, advanced pathological stage, or positive surgical margins. These findings highlight the need for risk-adapted follow-up and timely salvage treatment in high-risk LRP patients to improve long-term outcomes.

## 1. Introduction

Prostate cancer remains a significant global public health concern, with incidence and mortality varying based on screening practices, healthcare access, and awareness. In Europe, it is the most common male cancer, with approximately 341,000 new cases reported in 2020, accounting for 23% of all male cancer diagnoses [1]. Around 71,000 men died of prostate cancer that year, representing 10% of male cancer-related deaths. Variations in incidence rates, such as the high numbers reported in Ireland and France, likely reflect differences in screening and demographics.

In the United States, prostate cancer is projected to account for 299,010 new diagnoses in 2024 (14.9% of male cancer cases) and 35,250 deaths (11% of male cancer deaths) [2]. Increased awareness and early detection have contributed to a rising trend in incidence.

High-risk prostate cancer—characterized by elevated PSA, high Gleason score, or advanced tumor stage—is especially concerning due to its aggressive behavior and elevated risk of progression [3,4]. Despite advances in treatment, including laparoscopic radical prostatectomy (LRP) and external beam radiotherapy (EBRT), debate persists regarding the optimal management strategy [5,6]. Cryoablation is also rising as a promising modality [7]. 

Biochemical recurrence (BCR), defined as a postoperative rise in prostate-specific antigen (PSA) levels, is frequently the first indication of treatment failure [8]. Predictors of BCR include an initial PSA (iPSA) > 20 ng/mL, Gleason score, and positive surgical margins [9,10]. Some studies suggest that increased prostatic volume is also a risk factor [11]. Given the ongoing evolution in prostate cancer diagnostics and therapies, further research is required to refine treatment strategies, personalize risk stratification, and optimize long-term outcomes [12].

This study compares the oncological outcomes of LRP and EBRT combined with androgen deprivation therapy (ADT), with a focus on identifying predictors of BCR and assessing long-term recurrence-free, overall, and cancer-specific survival in patients with localized prostate cancer.

## 2. Subjects and Methods

A retrospective study was conducted at the Urology Department of St. Cyril and Methodius Hospital, University Hospital Bratislava, focusing on patients treated between 2016 and 2023. These patients had high-risk localized prostate cancer, as defined by EAU guidelines, and were treated by a single surgeon to minimize variability and reduce bias. Inclusion required meeting strict clinical and pathological criteria. All samples were collected in accordance with the principles outlined in the Declaration of Helsinki, following submission of informed consent. Patient data were anonymized. The study was approved by the Ethics Committee of the University Hospital Bratislava, St. Cyril and Methodius Hospital (EK 2/3/2023).

### 2.1. Selection Criteria

The staging workup included skeletal scintigraphy to exclude bone metastases, a whole-body 4-phase contrast-enhanced CT scan to assess lymph node and visceral involvement, and a 1.5-Tesla multiparametric MRI of the pelvis to determine the local stage of the disease. Patients were selected based on histological staging obtained from biopsy (coded as cTx stage), which may differ from the final pathological staging (coded as pTx stage) determined after LRP. The detailed inclusion and exclusion criteria are presented in Table 1.

### 2.2. Treatment Groups

Patients were assigned to one of two groups based on their choice of treatment:

1. LRP Group
  a. Underwent transperitoneal laparoscopic radical prostatectomy (TLRP);
  b. Extended pelvic lymphadenectomy using a modified Montsouris technique (Guillonneau et al., 2000) [14].
2. EBRT Group
  a. Received intensity-modulated radiation therapy (IMRT) to a total dose of 76 Gy;
  b. EBRT was initiated 4–6 months after ADT, and it was maintained for two years, keeping levels of circulating testosterone under 50 ng/L.

### 2.3. Monitored Parameters

At baseline, comprehensive diagnostic data were obtained for all patients, including iPSA, Gleason score from prostate biopsy, multiparametric MRI, CT scan, skeletal scintigraphy, and digital rectal examination.

Monitored parameters throughout the study included pre- and post-treatment PSA levels in both groups, as well as serum testosterone (TST) levels specifically in the EBRT group. PSA levels were assessed at each follow-up visit—every 3 months during the first 24 months and every 6 months thereafter, up to a maximum of 72 months (months 3, 6, 9, 12, 15, 18, 21, 24, 30, 36, 42, 48, 54, 60, 66, and 72). For patients followed at external centers, PSA results were obtained by telephone according to the same schedule. 

BCR was defined as a rise in serum PSA levels > 0.2 ng/mL in the LRP group and as an increase in PSA > 2 ng/mL plus nadir in the EBRT group [3]. In cases of suspected BCR, patients were referred back to the original urology department for further assessment, including restaging and consideration of initiating salvage therapy.

Monitored tumor characteristics and histopathology included: clinical stage (cT), Gleason score, and ISUP grade before treatment; pathological stage (pT) and final Gleason score post-LRP; the presence of positive surgical margins; extent of neurovascular bundle preservation; lymph node involvement; and need for salvage therapy. Due to the study design and radiation therapy principles, it was not feasible to determine the preservation of the neurovascular bundle, surgical margins, pT stage, or lymph node involvement in the EBRT group through histopathological examination.

### 2.4. Tumor Stage and Gleason Score Concordance

A comparison between clinical tumor stage (cT) and pathological tumor stage (pT) in the LRP group (n = 61) revealed a concordance rate of 77%. Despite multiparametric MRI being used for initial staging, 14 patients were upstaged after radical prostatectomy: 11 to pT3a and 3 to pT3b. These cases illustrate a diagnostic mismatch, as no extracapsular extension was suspected on preoperative imaging. Nevertheless, these patients were not excluded from the study, highlighting the limitations of imaging-based staging in accurately identifying locally advanced disease. The overall rate of T-stage discrepancy was 23%.

A similar analysis was conducted to assess Gleason score concordance between preoperative prostate biopsies and final histopathological evaluations of the prostatectomy specimens. The results showed an 82% agreement in Gleason scoring. However, in 18% of patients, the final Gleason score was upgraded or changed, reflecting sampling limitations inherent in needle biopsy. Most of the discordant cases involved an upgrade to a higher Gleason pattern, notably to 4 + 4 = 8 or 4 + 5 = 9.

### 2.5. Statistical Analysis

All statistical analyses were performed using IBM SPSS Statistics version 29 (Armonk, NY, USA). Categorical variables were analyzed using Pearson’s Chi-square or Fisher’s exact test as appropriate. Continuous variables were analyzed using non-parametric methods, including the Mann–Whitney U test and the Kruskal–Wallis test. Normality of distributions was assessed using the Shapiro–Wilk test.

Due to the differing definitions of BCR among study groups, direct incidence-based comparisons were limited. Therefore, treatment outcomes were primarily compared using time-to-event analysis. BCR-free survival was estimated using Kaplan–Meier curves, with log-rank tests used to compare survival distributions between the LRP and EBRT groups. Additional Kaplan–Meier analyses were stratified by ISUP grade and iPSA categories.

To identify independent predictors of BCR, multivariate Cox proportional hazards regression was performed. The model included treatment group (LRP vs. EBRT) as a covariate, along with ISUP grade, pathological stage, surgical margin status, and iPSA levels. Results were reported as hazard ratios (HR) with 95% confidence intervals (CI). A *p*-value < 0.05 was considered statistically significant for all analyses. 

To correct for multiple comparisons, Bonferroni correction was applied to subgroup analyses involving multiple categories, including ISUP grades (five categories) and iPSA groups (three categories). A power analysis was not conducted.

## 3. Results

In total, the study included 107 patients (61 in the LRP group and 46 in the EBRT group), with 100 patients completing the full five-year follow-up period. During the study, seven patients died, and no patients were excluded for other reasons. Subjects in the LRP group were slightly younger, with a median age of 64 years (range, 53–74), while subjects in the EBRT group had a median age of 67 years (range, 51–74). Mean iPSA was 15.41 ng/mL in the LRP group and 15.66 ng/mL in the EBRT group. The median follow-up period was 60 months (IQR: 24–72) for LRP and 66 months (IQR: 49.5–72) for EBRT.

BCR was defined differently for each treatment group, based on standard clinical criteria: PSA > 0.2 ng/mL for LRP and PSA rise > 2 ng/mL above nadir for EBRT [8]. Despite this difference, time-to-event analysis allowed comparison of recurrence-free survival across treatment modalities. Clinical outcomes based on study groups are presented in Table 2.

All patients in the LRP group who experienced BCR underwent salvage radiotherapy. Two of these patients subsequently progressed to metastatic disease and required systemic therapy. In the EBRT group, all patients with BCR underwent salvage LRP, and one progressed to metastasis during follow-up.

**Figure 1:**
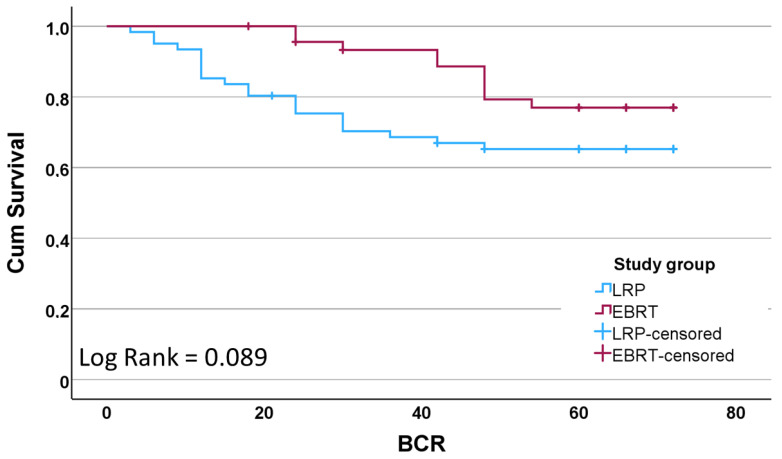
Comparison of BCR-free survival between study groups.

**Figure 2:**
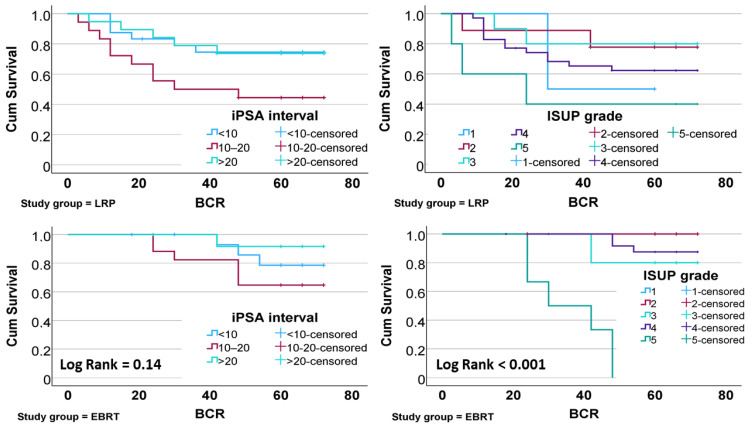
Comparison of BCR-free survival between study groups stratified by iPSA levels and ISUP grade.

BCR-free survival was compared between treatment groups using Kaplan–Meier analysis. As shown in Figure 1, EBRT demonstrated a higher BCR-free survival rate than LRP, with the difference approaching statistical significance (log-rank *p* = 0.089). When stratified by iPSA and ISUP grade, patients with intermediate iPSA levels (10–20 ng/mL) showed the shortest recurrence-free survival in both treatment groups (Figure 2). A similar trend was observed with higher ISUP grades (4–5), which were associated with reduced BCR-free survival (log-rank *p* < 0.001).

Predictive factors for BCR were further analyzed by treatment group (Table 3). In the LRP group, ISUP grade, pathological stage, and positive surgical margins were significantly associated with BCR (*p* < 0.001). iPSA showed a significant association with recurrence (*p* = 0.080). In contrast, no statistically significant predictors were identified in the EBRT group.

Multivariate analysis identified positive surgical margins and iPSA interval as independent predictors of recurrence in the LRP group. No independent predictors were identified for the EBRT group, potentially due to the lower number of events and smaller sample size.

Non-parametric tests revealed no significant difference in iPSA between treatment groups, recurrence status, age, or time to recurrence. However, iPSA levels were significantly higher in patients with higher ISUP scores (*p* < 0.001) and those with an advanced clinical stage (*p* = 0.048).

## 4. Discussion

Independent of the primary treatment modality, within 10 years, BCR occurs in 20–50% of patients with prostate cancer [15]. Published data report recurrence in 20–40% of patients following LRP and in 30–50% after EBRT [16,17]. In this study, 21 (34.4%) of subjects in LRP and 10 (21.7%) of those in EBRT experienced recurrence within the follow-up period, which aligns with globally reported data. Our findings notably highlight that positive surgical margins and higher ISUP grades are significantly predictive of recurrence following LRP. In contrast, intermediate PSA levels (10–20 ng/mL) unexpectedly showed higher recurrence rates compared to those with PSA levels greater than 20 ng/mL.

BCR-free survival progressively declines with the increasing number of risk factors [18]. A study on risk factors influencing BCR after surgical treatment of high-risk prostate cancers concluded that the presence of ≥2 risk factors, according to NCCN guidelines, is significantly associated with a higher BCR rate [19].

Prior research has consistently reported higher Gleason scores (or ISUP grades), advanced pathological stages, and positive surgical margins as significant predictors of biochemical recurrence post-prostatectomy. For instance, Tanimoto et al. conducted a prospective analysis involving 203 patients, demonstrating significant associations between positive margins, higher PSA levels, Gleason ≥ 8, and advanced pathological stages with recurrence [20]. Similarly, a multicenter retrospective study by Aoun et al. involving 1252 patients undergoing prostatectomy found high PSA and Gleason score ≥ 7 as independent predictors of recurrence, although this study exclusively included pathological stage pT2 patients with negative surgical margins [21]. Low- and intermediate-risk tumors indicate that the cT stage, Gleason score, and PSA levels are relevant factors for BCR [22]. 

In a study by Murata et al. (n = 376), seminal vesicle invasion, extraprostatic extension, and positive surgical margins were consistently identified as risk factors for BCR among high-risk patients treated surgically [23]. In studies by Kanehira et al. (n = 113) and Shindo et al. (n = 96), elevated preoperative PSA levels (>20 ng/mL), Gleason pattern 5, and positive surgical margins were identified as independent predictors of biochemical recurrence following radical prostatectomy in high-risk prostate cancer patients. Kanehira et al. additionally reported that perineural invasion and the presence of multiple adverse pathological features significantly reduced 2-year BCR-free survival, while Shindo et al. confirmed that high PSA levels were significantly associated with early recurrence, particularly in patients presenting with more than one high-risk feature [24,25]. John et al. conducted a systematic review and meta-analysis, finding that PSM length was independently associated with increased BCR risk, with margins > 3 mm conferring a higher risk (HR 1.99, 95% CI: 1.54–2.58), compared to shorter margins [26]. Beckmann et al. analyzed data from a community-based registry (n = 1221) and reported that patients with positive surgical margins had higher rates of BCR and subsequent treatments, emphasizing the clinical significance of margin status [27].

However, contrary to earlier investigations, this study found that individuals with an iPSA greater than 20 ng/mL did not exhibit the expected increase in BCR rates. The highest recurrence rates were in the iPSA group of 10–20 ng/mL, which aligns with a large database study from the USA [28]. Our findings indicate a lower BCR-free survival rate in the group with iPSA in the range of 10–20 ng/mL (*p* = 0.080). An interesting observation is that in the group of patients who underwent LRP with iPSA > 20 ng/mL, where, based on previous studies, a higher BCR rate could be expected. Our results diverge from the conclusions of these studies. Similar findings were also observed in the group of patients who underwent EBRT. 

The discrepancy regarding PSA levels could be attributed to patient selection bias in our study. Patients with higher PSA values, potentially associated with more aggressive disease, may have been inadvertently excluded due to stricter selection criteria aiming at localized, surgically treatable disease. Additionally, elevated PSA in some patients may reflect inflammation or benign prostate conditions rather than aggressive cancer, potentially confounding PSA-based risk stratification [29]. These limitations underscore the need for advanced multimodal prognostic systems that incorporate molecular biomarkers and imaging techniques alongside traditional risk factors to improve patient selection and prognostication accuracy, as well as profiling, advanced imaging technologies, and better risk stratification models [30].

Limitations of our study include its retrospective, single-center design, the relatively small sample size, and inherent selection bias resulting from patient-driven treatment choices. The small number of biochemical recurrence events particularly impacts the statistical power of multivariate analyses, cautioning against overgeneralizing our findings. Additionally, the different definitions of BCR applied to each treatment modality—PSA > 0.2 ng/mL for LRP and PSA nadir plus 2 ng/mL for EBRT—while reflective of real-world clinical practices, limit direct comparability between groups. Furthermore, only patients undergoing LRP provided histological specimens for margin evaluation, creating an inherent bias toward identifying positive surgical margins as predictive factors exclusively within the surgical group. This difference necessitates clarification, especially when interpreting surgical margin outcomes. 

Moreover, our cohort included a small subset of patients (9.8%) diagnosed as pT3 post-surgery, which raises the possibility of selection bias, as pathological staging was not uniformly available across both treatment arms. Future analyses should consider excluding these pT3 patients or conducting subgroup analyses to mitigate potential biases related to pathological staging discrepancies. Furthermore, it is essential to clarify explicitly in interpreting our results and conclusions that positive surgical margins are applicable only to the LRP-treated subgroup, whereas ISUP grading is applicable across the entire cohort. 

Despite these limitations, our study reinforces the prognostic importance of ISUP grading and positive surgical margins in predicting recurrence following prostatectomy. Our findings also challenge the conventional PSA risk categorization, suggesting that patients with intermediate PSA levels (10–20 ng/mL) may require more intensive surveillance and consideration for earlier intervention, even when traditional high-risk factors are not fully present.

## 5. Conclusions

This study confirms that BCR rates after LRP and EBRT in high-risk localized prostate cancer are consistent with international data. Recurrence occurred in 34.4% of LRP patients and 21.7% of EBRT patients over a five-year period. Multivariate analysis revealed that for the LRP group, positive surgical margins and ISUP grade were significant predictors of recurrence. At the same time, lymph node positivity showed a non-significant trend toward an increased risk. Interestingly, iPSA > 20 ng/mL did not predict higher recurrence, while the 10–20 ng/mL group had the highest BCR rates, echoing findings from recent large datasets.

Despite a higher BCR rate in LRP patients, no significant differences were found between groups in overall survival (OS) or cancer-specific survival (CSS). These findings suggest that relying solely on PSA for risk classification is inadequate and underscore the need for an integrated, multimodal risk stratification that incorporates molecular and imaging biomarkers. Future studies incorporating molecular and imaging biomarkers are warranted to improve risk classification and guide post-treatment surveillance strategies.

## Figures and Tables

**Table 1:** Inclusion and exclusion criteria.

Inclusion Criteria	Exclusion Criteria
Histologically confirmed localized prostate cancer	Locally advanced or metastatic disease (cT3-4, cN1, or cM1)
Male patients eligible for and consenting to curative treatment	PSA > 50 ng/mL [13]
Life expectancy > 10 years	Life expectancy < 10 years
Disease limited to the prostate (no metastases)	Prior curative treatment or contraindication to curative treatment
At least one of the following—clinical stage of up to cT2c, or initial PSA > 20 ng/mL, or Gleason score > 7	

**Table 2:** Summary of BCR and clinical outcomes.

Variable		LRP	EBRT
		N (%)	N (%)
Patients with BCR		21 (34.4)	10 (21.7)
5-year BCR-free survival		40 (65.6)	33 (71.7)
Median time to BCR		30.0 months (IQR: 12.5–60.0)	48.0 months (IQR: 30.0–60.0)
Neurovascular bundle preservation	none	25 (40.9)	-
	unilateral	32 (52.5)	-
	bilateral	4 (6.6)	-
Lymph node status	negative	55 (90.2)	-
	positive	6 (9.8)	-

**Table 3:** Association of clinical and pathological factors with BCR by treatment group.

Variable		LRP		EBRT	
		BCR (n)	BCR-Free (n)	BCR (n)	BCR-Free (n)
ISUP grade	1	1	1	0	1
	2	2	7	0	8
	3	2	8	1	4
	4	13	22	3	23
	5	3	2	6	2
χ^2^ *p*-value		<0.001		0.510	
Pathological stage	pT2	12	35	-	-
	pT3a	9	2	-	-
	pT3b	0	3	-	-
χ^2^ *p*-value		<0.001		-	
Surgical margins	Negative	11	38	-	-
	Positive	10	2	-	-
χ^2^ *p*-value		<0.001		-	
iPSA interval	<10	18	6	13	3
	10–20	8	10	11	6
	>20	14	5	12	1
χ^2^ *p*-value		0.080		0.180	

## Data Availability

The data is available from corresponding author based on a reasonable request.
